# Effects of Different Treatment Methods Used in Patients with Rheumatoid Arthritis on the Trabecular and Cortical Structure of the Mandible

**DOI:** 10.3390/diagnostics15030306

**Published:** 2025-01-28

**Authors:** Hatice Yemenoglu, Melek Beder, Osman Cüre, Dilara Nil Günaçar

**Affiliations:** 1 Department of Periodontology, Faculty of Dentistry, Recep Tayyip Erdoğan University, Rize 53020, Turkey; melek.beder@erdogan.edu.tr; 2 Rheumatology Subdivision, Department of Internal Diseases, Faculty of Medicine, Recep Tayyip Erdoğan University, Rize 53020, Turkey; osman.cure@erdogan.edu.tr; 3 Department of Oral and Maxillofacial Radiology, Faculty of Dentistry, Recep Tayyip Erdoğan University, Rize 53020, Turkey; dilaranil.tomrukcu@erdogan.edu.tr

**Keywords:** fractal analysis, rheumatoid arthritis, TNF-α inhibitor, DMARDs, glucocorticoid

## Abstract

**Background/Objectives:** To appraise the mandibular bone structure of patients with rheumatoid arthritis (RA) who were treated with different drugs using fractal dimension (FD) analysis and panoramic radiomorphometric indices and investigate the effects of RA on the jaw bone. **Methods:** A total of 90 panoramic radiographs were analyzed in this study: 30 were from patients with RA using conventional synthetic disease-modifying antirheumatic drugs (csDMARDs) and glucocorticoids, 30 were from patients with RA using tumor necrosis factor-alpha (TNF-α) inhibitors, and 30 were from systemically healthy individuals. In panoramic radiographs, panoramic mandibular index (PMI), mandibular cortical thickness measurements, mandibular cortical index (MCI), and FD analysis taken from four different regions were evaluated. **Results:** The lowest FD analysis value was observed in patients using csDMARDs and glucocorticoids and in the condyle region (*p* = 0.001). Although PMI and posterior index (PI) were found to be significantly lower in patients using csDMARDs and glucocorticoids compared with the others (*p* = 0.001), no significant difference was observed between the groups in terms of anterior index (AI), symphysis index (SI), and molar index (MI) values (*p* > 0.05). When MCI was analyzed, there was no significant difference between the groups (*p* > 0.05). **Conclusions:** It was observed that the trabecular structure in the condyle and posterior region of the mandible was negatively influenced by RA and csDMARD and glucocorticoid drug use. Radiomorphometric indices and FD analysis on panoramic radiographs can be used to evaluate osteoporotic alterations in individuals with RA. These assessments are valuable in predicting the prognosis of conditions such as bone healing after tooth extraction and other surgical procedures and osseointegration after implant surgery.

## 1. Introduction

Rheumatoid arthritis (RA) is a chronic, multifactorial, inflammatory disease of unknown etiology that affects the joints [1]. It affects approximately 1% of adults and is mostly seen in women [2]. Temporomandibular joint (TMJ) damage is generally observed in advanced conditions [3]. Radiologically, changes such as synovial cysts, loss of bone mass, bone erosion, reduced bone density in the bone adjacent to the joint, and loss of articular cartilage are observed [1].

Because inflammation is the basis of clinical findings in RA, the main goal of treatment is to reverse this condition. Therefore, RA treatment requires continuous assessment of disease activity and necessary changes in medications according to this activity [4]. According to the European League Against Rheumatism (EULAR), it is recommended to start disease-modifying antirheumatoid drug (DMARD) treatment as soon as RA is diagnosed [5]. DMARDs are classified as conventional synthetic DMARDs (leflunomide, methotrexate, hydroxychloroquine, sulfasalazine), targeted synthetic DMARDs and biologic DMARDs (divided into several classes: TNF-α inhibitors (certolizumab, adalimumab, etanercept, infliximab, golimumab), IL-6 inhibitors (tocilizumab, sarilumab, etc.), B-cell receptor inhibitors (rituximab), janus kinase inhibitors (tofacitinib, upadacitinib), inhibitor of T cell costimulation (abatacept), etc.) [6]. Glucocorticoids are used for a short time while starting conventional synthetic DMARD treatment, which is recommended as the first step, and/or while switching between them. If there is no response to this treatment within 3–6 months, targeted synthetic or biologic DMARDs are started depending on the presence of poor prognostic factors [5].

Methotrexates, which are in the conventional synthetic DMARDs (csDMARDs) group, are among the first-line drugs used in the treatment of RA. In a study, increased RANKL and decreased OPG/RANKL ratios were determined in individuals with RA before methotrexate treatment compared with the healthy group, whereas an increase in the OPG/RANKL ratio was observed after at least 6 months of methotrexate treatment [2]. In addition, it has been reported that the use of methotrexate and low-dose prednisone reduces the soluble RANKL/OPG ratio [7]. It has been reported that methotrexate protects bones by regulating RANKL/RANK/OPG signaling [8].

Glucocorticoids are widely utilized in the therapy of inflammatory diseases due to their robust immunosuppressive and anti-inflammatory effects. However, glucocorticoid-induced osteoporosis represents the most prevalent cause of secondary osteoporosis. High concentrations of glucocorticoids significantly diminish the number and activity of osteoblasts, reduce bone formation rates, and diminish the number of osteocytes. Additionally, a rapid decline in bone mineral density is one of the immediate impacts of glucocorticoid use [9]. While several studies propose that TNF-α inhibitors positively influence bone mineral density [10,11,12], Kawai et al. [13] reported that TNF-α inhibitors do not prevent bone loss.

Fractal dimension (FD) analysis is a measurement technique that employs the box-counting algorithm to evaluate complex structures, such as the trabecular bone. Higher FD values correspond directly to increased complexity of bone structure [14]. In dentistry, FD analysis is often used to assess and quantify bone mineral density, changes in periapical bone, apical healing, and the impact of systemic diseases or medications on the jawbone [15]. Radiomorphometric indices, including the mandibular cortical index (MCI), panoramic mandibular index (PMI), and mandibular cortical width (MCW), are used to identify bone quality parameters such as cortical bone thickness, the degree of bone mineralization, trabecular bone structure, osteoporosis, and osteopenia [16].

To the best of our knowledge, no prior study has researched the effects of TNF-α inhibitors on the mandibular bone structure utilizing FD analysis. This study purposed to assess the impact of TNF-α inhibitors on the mandible in RA patients by analyzing FD and panoramic morphometric indices, such as mandibular cortical thickness measurements, the PMI, and the Klemetti index (KI). The findings were compared with those of RA patients who had not used TNF-α inhibitors and a control group of systemically healthy individuals.

## 2. Materials and Methods

### 2.1. Study Groups

This study was administered retrospectively, adhering strictly to valid ethical principles, including the World Medical Association Declaration of Helsinki 1964 and its subsequent amendments. Ethical approval was received from the Recep Tayyip Erdoğan University Ethics Committee (Decision no: 2024/250). This study’s purpose and content were clearly explained to all participants, and informed consent was received from each patient. Data were collected retrospectively by reviewing the anamnesis and digital panoramic radiographs of patients aged 45–60 years who visited the Faculty of Dentistry, Recep Tayyip Erdoğan University for various symptoms between January 2022 and August 2024.

Information on systemic health and medication use was gathered from the patients’ medical records. Ninety individuals were enrolled in the research, separated equally into three groups of 30 participants each. Group 1 consisted of RA patients treated with csDMARDs and glucocorticoids for at least one year (csDMARD + glucocorticoid group), Group 2 included RA patients who had been using TNF-α inhibitors for at least one year (TNF-α inhibitor group), and Group 3 comprised systemically healthy individuals with no history of medications affecting bone metabolism (control group). Participants for the study groups were randomly selected using digital software (available at: www.randomizer.org accessed on 9 October 2024).

Inclusion criteria were defined as postmenopausal female patients aged 45–60 years, possessing panoramic radiographs of sufficient diagnostic quality to allow the evaluation of cortical and trabecular structures, showing no intraosseous pathology in the regions designated for FD analysis, and without osteoporosis/osteopenia diagnosis based on Dual Energy X-ray Absorptiometry (DEXA) values. The age ranges of the participants in the control and study groups were paired to ensure compatibility.

Exclusion criteria included patients taking medications known to affect bone density (except for TNF-α inhibitors, DMARDs, and glucocorticoids), those with conditions that influence bone density (e.g., osteomalacia, diabetes, kidney diseases, hypothyroidism, hyperparathyroidism), or mandibular pathologies such as cystic tumors. Also excluded were patients with osteoarthritic changes in TMJ by radiographic examination and/or symptoms or complaints related to TMJ in clinical examination, panoramic radiographs of insufficient diagnostic quality, and participants diagnosed with osteoporosis/osteopenia based on DEXA values. Previous studies have shown that the Helkimo Clinical Dysfunction Index (HCDI) is able to detect temporomandibular joint disorders (TMD)-affected subjects with rheumatoid arthritis, with a statistically significant difference between affected and unaffected subjects [17,18,19]. We also used HCDI to evaluate the TMJ damage of our patients, and the TMJ involvement score of our patients was 0 point. Individuals with advanced periodontal disease (stage 3–4 periodontitis) and those missing more than one anterior or posterior tooth were also excluded to standardize the evaluation of the trabecular structure and to avoid masticatory dysfunction as a confounding factor.

The RA disease activity status of the patients was determined according to the Disease Activity Score (DAS) 28-erythrocyte sedimentation rate (ESR). According to the DAS 28-ESR scores, the patients were evaluated as in remission (≤2.6), low (2.6 between ≤3.2), moderate (>3.2 between ≤5.1), and high disease activity (>5.1) phases [20].

Panoramic radiographs for all participants were obtained using standardized exposure settings (66 kVp, 8 mA, 16.6 s) with the same equipment (Planmeca Promax 2D S2, Planmeca Oy, Helsinki, Finland). Patient positioning adhered to the manufacturer’s guidelines, ensuring the Frankfurt plane was parallel to the floor and the sagittal plane aligned with the vertical plane of the panoramic machine.

### 2.2. Measurements

#### 2.2.1. Fractal Dimension Analysis

**Figure 1:**
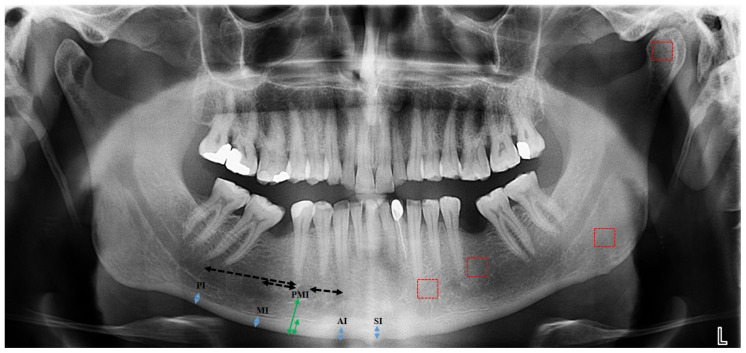
Region of interest areas (ROIs) selected for fractal analysis in the left hemimandible are shown: the subcortical area in the condyle, the supracortical area above the angle of the mandible, above the mandibular canal distal side to the second premolar, anterior to the mental foramen (red boxes). Radiomorphometric measurements (blue arrows) are shown in the right hemimandible: SI; symphysis index, AI; anterior index, MI; molar index, PI; posterior index, PMI (green arrows); panoramic mandibular index.

The images were examined using Image J v. 1.52 software (National Institutes of Health, Bethesda, MD, USA), freely available for download at https://imagej.nih.gov/ij/download.html. (accessed on 1 October 2024) Measurements were obtained for each patient by selecting 45 × 45-pixel regions of interest (ROIs) on both the right and left sides of panoramic radiographs using the box-counting method [21]. The mean FD value was calculated by averaging the measurements from both sides. The evaluated regions included the subcortical area of the condyle (FD1), the supracortical region above the mandibular angle (FD2), the mandibular canal adjacent to the second premolar (FD3), and the anterior region of the mental foramen (FD4), all located within the trabecular bone (Figure 1).

**Figure 2:**
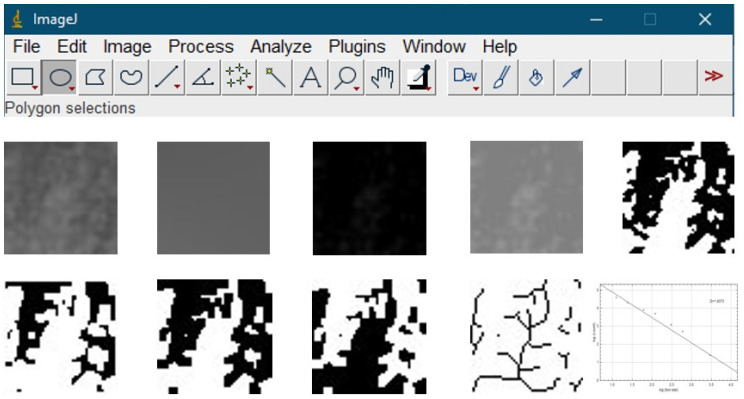
Demonstration of fractal analysis process steps: (**top row**); image J program, (**middle row**) respectively, cropped image, Gaussian blur filtered image, subtracted image, added image, threshold applied image, (**bottom row**) respectively: eroded image, dilated image, inverted image, skeletonize image, box-counting method result.

To optimize image quality, panoramic radiographs were transformed into labeled image file formats. A total of 480 ROIs were chosen, clipped, and duplicated for analysis. Gaussian Blur was applied to reduce brightness inconsistencies due to superimposed soft tissues or altered bone thickness. The committed image was then extracted from the original image. Trabecular structures and bone marrow gaps were isolated by applying 128 Gy to each pixel. Subsequent steps, including binarization, erosion, dilation, inversion, and skeletonization, were performed to prepare the images for fractal analysis. The FD was calculated by separating the pixel squares into units involving trabecular structures and determining the number of total frames at different pixel dimensions (Figure 2).

#### 2.2.2. Radiomorphometric Analysis

Radiomorphometric evaluations were measured using Planmeca Romexis 4.6.2.R software (PLANMECA Romexis, Helsinki, Finland). Cortical thickness measurements, enforced by Barra et al. [22] using cone beam computed tomography (CT), were adapted for panoramic radiograph analysis. Morphometric indices were assessed individually in the posterior, molar, anterior, and symphysis regions on both the left and right sides, and the average value was calculated from these measurements. The posterior index (PI) was determined to be 2.5 cm posterior to the mental foramen, vertical to the inferior cortex, and parallel to the mandibular long axis. The molar index (MI) was obtained 1 cm posterior to the mental foramen, following a similar perpendicular and parallel alignment. The anterior index (AI) was determined as 1 cm anterior to the mental foramen, vertical to the inferior mandibular cortex, and aligned with the mandible long axis. The symphysis index (SI) was calculated perpendicular to the inferior border of the mandibular cortex, at equal distances from the centers of the left and right mental foramen (Figure 1) [22].

The MCW was calculated as the perpendicular space from the lower boundary of the mandible at the midpoint of the mental foramen. The PMI was computed by dividing the MCW by the space between the inferior mandibular cortex and the mental foramen (Figure 1). This region is considered ideal for PMI measurements due to the lack of masticatory muscles near the mental foramen [23]. The mean PMI value was derived from the average of the right and left measurements.

**Figure 3:**
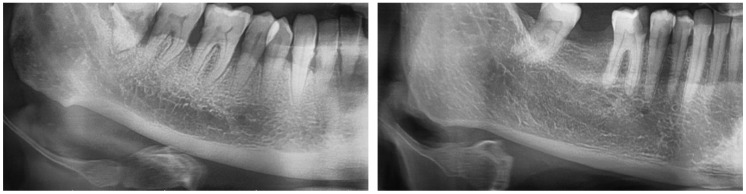
Klemetti index images: (**Left**) (normal cortex: the endosteal margin of the cortex is matched and tapered) and (**Right**) (moderately eroded cortex: endosteal margin, lacunar resorption resulting in semilunar defects or formation of endosteal cortical residues) respectively.

The MCI, also known as KI, is utilized to evaluate the quality of the subcortical bone behind the mental foramen. The qualitative index is divided into three classes: C1 (normal cortex)—characterized by a continuous and smooth endosteal edge; C2 (moderately eroded cortex)—defined by lacunar resorption forming half-moon-shaped defects or remnants of endosteal cortical bone; and C3 (severely porous cortex)—defined by large porosity with dense cortical residues (Figure 3) [24].

All assessments were conducted by an expert oral and maxillofacial radiologist (D.N.G.), who was blinded to the clinical data of the participants. To ensure intraobserver credibility, 20% of the panoramic images were selected randomly and reassessed one month after the initial evaluation.

#### 2.2.3. Statistical Analysis

For measurements, the Shapiro–Wilk test demonstrated that the data fit the normal distribution. The descriptive statistics were described as minimum, maximum, standard deviation (SD), and mean, and frequency distributions (number and percentage). A one-way analysis of variance (ANOVA) was applied to comparing the measurements across the three groups. Group differences were then analyzed using the post hoc Tukey test. The associations between the groups and the KI were assessed through Pearson’s Chi-square test. Pearson correlation coefficients were calculated to determine the relationships between continuous measurements. *p* < 0.05 was determined as a statistically significant value. Intraobserver agreement was assessed by intraclass correlation coefficient (ICC) calculation. For all statistical analyses, SPSS software (version 23) was used.

##### Sample Size Calculation

As a result of the power analysis, it was identified that a minimum of 90 participants would be required for a three-group study. The analysis assumed an effect size of 0.53%, a confidence interval of 95%, and a statistical power of at least 95%. Accordingly, the total number of participants and the distribution among the groups were established (G*Power 3.1.0 software package, Universitat Düsseldorf, Düsseldorf, Germany).

## 3. Results

This study enrolled a total of 90 participants, divided into three groups: 30 patients with RA receiving csDMARDs and glucocorticoids, 30 patients with RA treated with TNF-α inhibitors, and 30 healthy individuals. The participants’ mean age was 51.4 ± 6.05 years, with a range of 45–60 years. The mean ages for the csDMARD and glucocorticoid group, TNF-α inhibitor group, and control group were 50.4 ± 6.9, 51.3 ± 5.7, and 52.5 ± 5.4 years, respectively, with no statistically significant differences between the groups (*p* = 0.409). All participants were female. Measurements from the left and right hemispheres of each patient’s panoramic radiographs were averaged for comparisons.

The findings of the FD analysis across four different ROIs in each hemisphere are presented in Table 1. Significant differences in FD values were identified among the groups (*p* = 0.001). The least FD values were observed in the csDMARD + glucocorticoid group, while the control group exhibited the highest FD values. Specifically, FD1 was significantly reduced in the csDMARD + glucocorticoid group, whereas the control group displayed the highest FD1 value (*p* = 0.001). Control groups’ FD2 measurements were significantly greater than those in the TNF-α inhibitor group and the csDMARD + glucocorticoid group (*p* = 0.001), though no significant differences observed between the latter two groups (*p* > 0.05). In regard to FD3, the TNF-α inhibitor group showed significantly lower values as opposed to the control group (*p* = 0.001), with no notable differences between other groups (*p* > 0.05). FD4 values were significantly reduced in both the csDMARD + glucocorticoid and TNF-α inhibitor groups when opposed to the control group (*p* = 0.001).

Intragroup comparisons showed that the mean FD1 was lower than those of FD2, FD3, and FD4 in the csDMARD + glucocorticoid and TNF-α inhibitor groups (*p* < 0.001). In these groups, there was no significant difference between FD2, FD3, and FD4 (*p* > 0.05). In the control group, there was no meaningful difference between the average FD values among all regions (*p* > 0.05) (Table 1).

Table 2 presents the cortical thickness measurements. csDMARD + glucocorticoid group had an average PI that was significantly lower in comparison with both the TNF-α inhibitor group and the control group (*p* < 0.05). No meaningful differences were detected in the mean AI, MI, and SI values across groups (*p* > 0.05). The mean PMI was significantly lower in the csDMARD + glucocorticoid group than in the remaining two groups (*p* < 0.001), but no significant differences were observed between the TNF-α inhibitor group and the control group (*p* > 0.05). Within-group comparisons showed that posterior thickness was consistently lower than other regions in all three groups, while symphysis thickness demonstrated one of the highest mean values (*p* < 0.001).

Detailed regional analysis indicated that in the csDMARD + glucocorticoid group, PI showed the lowest values (*p* < 0.001), while SI displayed the highest (*p* < 0.001). No significant differences were found between PMI, AI, and MI. In the TNF-α inhibitor group, PI had the lowest values, whereas SI, PMI, and AI had the highest averages, with MI differing significantly from the others (*p* < 0.001). In the control group, PI was the lowest, and SI and PMI had the highest mean values (*p* < 0.001). Table 3 summarizes the distribution of KI values among the groups. Most participants had a KI value of 1, and no individuals had a KI of 3. Klemetti indices were not significantly different between the groups.

As shown in Table 4, the concordance between the first and second assessments of FD, cortical thickness, and PMI values was highly consistent. The lowest ICC values exceeded 0.90, indicating excellent reliability [25].

There was no significant difference between the csDMARD + glucocorticoid group and the TNF-α inhibitor group in terms of DAS 28-ESR values (*p* > 0.05). When the disease activity of RA patients was evaluated according to DAS 28-ESR, 50% of the patients in the csDMARD + glucocorticoid group had moderate disease activity, while 50% were in remission. In the TNF-α inhibitor group, 10% of the patients had low disease activity, 33% had moderate disease activity, and 57% were in remission.

When the correlation between disease activity and FD analysis values and radiomorphometric indices was examined, there was a statistically significant negative correlation between DAS 28-ESR and the FD value in the condyle region of patients using csDMARDs + glucocorticoids (r = −0.405), no significant correlation was observed between DAS 28-ESR and other parameters (Table 5).

## 4. Discussion

To our knowledge, the present study is the first to investigate the effects of different therapy methods on mandibular bone structure in RA patients utilizing radiomorphometric indices and FD analysis. The findings demonstrated that the control group had the highest FD values, while the group treated with csDMARDs and glucocorticoids exhibited the lowest FD values, particularly in the condyle region. Although the mean values for the PI and PMI in the csDMARD and glucocorticoid groups were significantly lower compared to the other groups, no significant differences were identified for the AI, MI, SI, or KI. Additionally, most participants across all groups had KI values categorized as 1.

FD has been identified as a useful parameter for distinguishing osteoporotic changes from normal bone density [26]. Previous research has found significantly lower FD values in individuals using intravenous corticosteroids [16], aromatase inhibitors [27], antiepileptic drugs [28,29,30], selective serotonin reuptake inhibitors (SSRIs) [31], and in those diagnosed with conditions such as sickle cell anemia [32,33], osteoporosis [34], chronic renal failure [35], thalassemia major [36], psoriasis vulgaris [37], ankylosing spondylitis [38], hyperparathyroidism [39], and vitamin D deficiency [40] compared to control groups. Conversely, research involving bisphosphonate users found lower FD values in the healthy subjects than in the drug group, likely causing decreased bone resorption in patients treated with bisphosphonates [41]. Other studies on patients with celiac disease [42] and those taking oral anticoagulants [43] found no important differences in FD measurements between groups. In systemic glucocorticoid users, FD measurements in cancellous areas near the ramus, angulus, and mental foramen showed no significant differences, although FD values in the basal corpus distal to the mandibular first molar were significantly lower than in controls [9]. Similarly, Günaçar et al. [14] observed lower FD values in hyperlipidemia patients compared to controls, with only the FD value at the mandibular angle being significantly reduced.

In our study, significant differences in FD values were observed across groups. The lowest FD value was recorded in the condyle region (FD1) of patients treated with csDMARDs and glucocorticoids, while the highest FD value was found in the anterior region (FD4) of the control group. This may be related to the density loss observed in bones adjacent to the joint in RA patients, as well as the negative impact of glucocorticoids and csDMARDs on bone metabolism. Intragroup comparisons revealed that FD values in the condyle region were significantly lower than those in other regions among patients treated with csDMARD + glucocorticoids and TNF-α inhibitors. However, regional differences were not found in the control group. This may reflect the effects of TMJ damage in RA patients and the associated reduction in bone density in areas adjacent to the joint.

It is known that cortical bone thickness varies in different regions of the bone for both the maxilla and mandible in various age groups [44,45]. Due to the clinical importance of evaluating cortical bone thickness in dental implant treatment planning, this study aimed to investigate the effects of different drug groups used to treat RA on cortical bone in patients aged 45–60 years. Temple et al. [45] stated in their study that buccal cortical bone thickness tends to decrease apico-coronally in the maxilla and increase apico-coronally in the mandible. However, they also found a general tendency for both jaws to increase in thickness from front to back [45]. It has been reported that panoramic radiomorphometric indices can be utilized as a preliminary diagnostic tool to determine cortical bone osteoporotic changes [26]. When we look at the literature, there are studies in which bone mineral density is evaluated with measurements such as MCW, MCI, and PMI in panoramic radiographs [35,41,46,47]. Many studies have found that MCW values are lower in individuals with osteoporosis as opposed to healthy individuals [21,35,47,48,49,50]. In the present study, instead of MCW, the four new radiomorphometric indices PI, MI, AI, and SI used by Barra et al. [22] were used to assess bone mineral density. Based on our findings, there was no statistically significant difference between the groups in SI, AI, and MI values, whereas PI value was significantly lower in csDMARDs + glucocorticoids users as opposed to TNF-α inhibitors users and healthy subjects, whereas in the other two groups, no significant difference was found in PI value. This may be due to the negative effects of csDMARDs and glucocorticoids on bone metabolism and bone mineral density and the positive efficacies of TNF-α inhibitors on bone mineral density. When the within-group evaluations were examined, the PI score was significantly lower than the other indices. In studies conducted on individuals with hyperlipidemia [14] and vitamin D deficiency [40], it was reported that there was no statistically significant difference between the groups in PI, MI, AI, and SI parameters, and when the within-group evaluations were examined, PI values were significantly lower than other parameters, similar to our findings. In addition, Barra et al. [22] found that MI and PI can be a useful tool for determining low bone mineral density in postmenopausal patients.

The findings of this study revealed that the PMI value was significantly lower in the csDMARD + glucocorticoid group as opposed to the other groups. This result aligns with previous research indicating that PMI values are significantly reduced in individuals with vitamin D deficiency [40], as well as in those taking oral anticoagulants [43], antiepileptic medications [28], and aromatase inhibitors [27], as opposed to healthy individuals. But, contrary to our results, some studies have reported no important differences in PMI values between groups [9,14,16,31,36,42].

Our analysis also found no statistically significant differences in MCI measurements between the groups. This observation is consistent with studies that found no significant group differences in MCI values [9,27,28,31,36,42,43]. In contrast, some studies have reported significant differences in MCI values between groups [14,40]. These discrepancies may stem from variations in the drugs or systemic conditions under investigation, and the qualitative nature of the MCI assessment. In our study, most participants across all three groups had a cortical index classified as C1. This finding may be attributed to the higher degree of mineralization and stiffness in cortical bone compared to trabecular bone, which makes cortical bone less susceptible to the effects of medications.

In addition, in this study, a negative correlation was observed between the FD value in the condyle region and RA disease activity in patients using csDMARD + glucocorticoids. There was no correlation between other FD measurement values and radiomorphometric index measurements and disease activity. This may be due to the effects of TMJ damage and the density loss observed in the bones adjacent to the joint in RA patients, as well as the negative effects of glucocorticoids and csDMARDs on bone metabolism.

There are some limitations to our study. First, because it was a retrospective study, there were patients who could not be included in this study due to incomplete anamnesis information. Second, in this study, only RA patients using glucocorticoids + csDMARDs and TNF-α inhibitor drug groups were included. In addition, when bone density may vary according to the duration of treatment, dosage, and patients’ compliance with treatment, these conditions should also be taken into account in future studies as they may affect the results of this study. We believe that long-term prospective studies that include more patients in different age groups, including subgroups in each drug group, can further improve our knowledge.

## 5. Conclusions

Based on the findings of the current study, the lowest FD value was found in individuals using csDMARDs and glucocorticoids and in the condyle region, the average PI and PMI of the csDMARDs and glucocorticoid users were significantly lower than the other participants, there was no significant difference between the groups in terms of AI, MI, SI, and KI, and the majority of KI values in the groups were C1. In addition, there was a negative correlation between the FD value in the condyle region and RA disease activity in those using csDMARDs and glucocorticoids. Radiomorphometric index measurements and FD analysis using panoramic radiographs can be used to evaluate osteoporotic changes in individuals with RA. In patients with RA disease that may have negative effects on the bone and especially in individuals with a history of csDMARD and glucocorticoid use, it may be more suitable to have these effects taken into account in estimating the prognosis for situations such as bone healing following tooth extraction or periodontal surgery, osteointegration after implant application, and complications that may occur afterwards.

## Figures and Tables

**Table 1:** Descriptive values of the fractal dimension measurements in this study and healthy groups.

		csDMARDs and Glucocorticoids							TNF-α Inhibitor							Control							
		Mean	SD	Min	Max	Percentiles			Mean	SD	Min	Max	Percentiles			Mean	SD	Min	Max	Percentiles			
	*n*					25th	Median	75th					25th	Median	75th					25th	Median	75th	*p* *
**FD1**	30	1.303 ^a,A^	0.016	1.273	1.340	1.291	1.302	1.315	1.327 ^b,A^	0.038	1.244	1.382	1.292	1.328	1.358	1.377 ^c^	0.010	1.360	1.394	1.369	1.376	1.386	**0.001**
**FD2**	30	1.356 ^a,B^	0.024	1.314	1.399	1.335	1.354	1.375	1.349 ^a,B^	0.036	1.276	1.406	1.328	1.349	1.384	1.376 ^b^	0.012	1.355	1.401	1.366	1.375	1.387	**0.001**
**FD3**	30	1.366 ^a,b,B^	0.024	1.301	1.403	1.353	1.368	1.385	1.353 ^b,B^	0.032	1.264	1.403	1.338	1.354	1.373	1.377 ^a^	0.013	1.347	1.408	1.367	1.376	1.385	**0.001**
**FD4**	30	1.361 ^a,B^	0.023	1.312	1.404	1.348	1.365	1.377	1.361 ^a,B^	0.032	1.285	1.415	1.345	1.365	1.386	1.382 ^b^	0.014	1.358	1.410	1.372	1.384	1.393	**0.001**
*p* **		**0.001**							**0.001**							0.135							

FD1, the subcortical area in the condyle; FD2, the supracortical area above the angle of the mandible; FD3, above the mandibular canal distal side to the second premolar; FD4, anterior to the mental foramen; SD, standard deviation; Min, minimum; Max, maximum; *n*, number; *, significance levels according to one-way ANOVA and post hoc Scheffe test results; **, significance levels in within-group evaluation; a, b, c, shows the difference between groups; A, B, shows differences between regions in the group.

**Table 2:** Descriptive values of the cortical thickness measurements in this study and healthy groups.

		csDMARDs and Glucocorticoids							TNF-α Inhibitor							Control							
		Mean *	SD	Min	Max	Percentiles			Mean *	SD	Min	Max	Percentiles			Mean *	SD	Min	Max	Percentiles			
	*n*					25th	Median	75th					25th	Median	75th					25th	Median	75th	*p* *
**SI**	30	0.329 ^C^	0.027	0.270	0.380	0.310	0.330	0.350	0.323 ^C^	0.022	0.280	0.360	0.310	0.325	0.340	0.324 ^D^	0.016	0.290	0.350	0.310	0.330	0.340	0.489
**AI**	30	0.314 ^B^	0.029	0.245	0.370	0.289	0.320	0.335	0.315 ^C^	0.020	0.270	0.345	0.299	0.318	0.330	0.311 ^C^	0.016	0.265	0.345	0.300	0.310	0.325	0.800
**MI**	30	0.300 ^B^	0.027	0.240	0.365	0.275	0.305	0.320	0.305 ^B^	0.020	0.270	0.345	0.290	0.305	0.320	0.302 ^B^	0.016	0.265	0.345	0.290	0.300	0.315	0.662
**PI**	30	0.282 ^a,A^	0.026	0.235	0.335	0.260	0.275	0.310	0.294 ^b,A^	0.019	0.260	0.335	0.284	0.290	0.310	0.292 ^b,A^	0.014	0.250	0.325	0.285	0.295	0.300	**0.050**
**PMI**	30	0.312 ^a,B^	0.022	0.261	0.351	0.304	0.319	0.326	0.324 ^b,C^	0.013	0.295	0.344	0.317	0.325	0.333	0.330 ^b,D^	0.010	0.306	0.344	0.322	0.331	0.339	**0.001**
*p* **		**0.001**							**0.001**							**0.001**							

SI, symphysis index; AI, anterior index; MI, molar index; PI, posterior index; PMI, panoramic mandibular index; SD, standard deviation; Min, minimum; Max, maximum; *, significance levels according to one-way ANOVA and post hoc Scheffe test results; **, significance levels in within-group evaluation; a, b, shows the difference between groups; A, B, C, D, shows differences between regions in the group.

**Table 3:** Distribution of Klemetti index results according to groups.

		csDMARDs and Glucocorticoids		TNF-α Inhibitor		Control		Total	
		*n*	%	*n*	%	*n*	%	*n*	*p*
**Klemetti Index**	**C1**	25	83.3	27	90.0	28	93.3	80	0.455
	**C2**	5	16.7	3	10.0	2	6.7	10	
**Total**		30	100.0	30	100.0	30	100.0	90	

Pearson chi-square test; *n*, number; C1, Klemetti index 1; C2, Klemetti index 2.

**Table 4:** Intraobserver agreement of the first and second measurements from region of interests.

	csDMARDs and Glucocorticoids				TNF-α Inhibitor				Control			
	ICC	95%CI		*p*	ICC	95%CI		*p*	ICC	95%CI		*p*
		Lower	Upper			Lower	Upper			Lower	Upper	
**FD1**	0.990	0.938	0.999	**0.000**	0.985	0.909	0.998	**0.000**	0.988	0.928	0.998	**0.000**
**FD2**	0.986	0.915	0.998	**0.000**	0.992	0.950	0.999	**0.000**	0.956	0.738	0.994	**0.001**
**FD3**	0.993	0.956	0.999	**0.000**	0.997	0.980	1.000	**0.000**	0.977	0.862	0.997	**0.000**
**FD4**	0.990	0.943	0.999	**0.000**	0.957	0.743	0.994	**0.001**	0.987	0.923	0.998	**0.000**
**Symphysis index**	0.972	0.830	0.996	**0.001**	0.928	0.570	0.990	0.003	0.933	0.601	0.990	**0.002**
**Anterior index**	0.981	0.886	0.997	**0.000**	0.974	0.845	0.996	**0.000**	0.969	0.813	0.996	**0.000**
**Molar index**	0.993	0.956	0.999	**0.000**	0.981	0.886	0.997	**0.000**	0.977	0.863	0.997	**0.000**
**Posterior index**	0.992	0.951	0.999	**0.000**	0.973	0.837	0.996	**0.000**	0.982	0.895	0.997	**0.000**
**PMI**	0.998	0.990	1.000	**0.000**	0.813	−0.119	0.973	**0.032**	0.990	0.942	0.999	**0.000**

FD1, the subcortical area in the condyle; FD2, the supracortical area above the angle of the mandible; FD3, above the mandibular canal distal side to the second premolar; FD4, anterior to the mental foramen; PMI, panoramic mandibular index; CI, confidence interval; ICC: intraclass correlation coefficient.

**Table 5:** Correlation between RA disease activity and FD analysis values and radiomorphometric measurements.

		csDMARDs and Glucocorticoids (*n* = 30)	TNF-α Inhibitor (*n* = 30)
		DAS 28-ESR	DAS 28-ESR
**FD1**	**r**	−0.405 *	−0.144
**FD2**	**r**	0.198	0.055
**FD3**	**r**	−0.246	−0.150
**FD4**	**r**	0.185	−0.130
**SI**	**r**	−0.282	0.161
**AI**	**r**	−0.237	0.040
**MI**	**r**	−0.242	0.051
**PI**	**r**	−0.254	0.080
**PMI**	**r**	−0.282	0.234

FD1, the subcortical area in the condyle; FD2, the supracortical area above the angle of the mandible; FD3, above the mandibular canal distal side to the second premolar; FD4, anterior to the mental foramen; SI, symphysis index; AI, anterior index; MI, molar index; PI, posterior index; PMI, panoramic mandibular index; DAS 28-ESR, Disease Activity Score 28-erythrocyte sedimentation rate; *n*, number; r, Pearson correlation coefficients; *, *p* < 0.05.

## Data Availability

There are no additional data deposited on any other site other than in this manuscript.
